# Circadian rhythm in platelet: an emerging therapeutic target in human diseases

**DOI:** 10.3389/fphys.2026.1795526

**Published:** 2026-09-04

**Authors:** Zihui Wang, Zhe Chen, Fei Li, Linxi Chen, Guozuo Xiong

**Affiliations:** 1 Department of Vascular Surgery, The Second Affiliated Hospital of University of South China, Hengyang, Hunan, China; 2 Institute of Pharmacy and Pharmacology, College of Basic Medical Science, University of South China, Hengyang, Hunan, China; 3 Hubei Key Laboratory of Wudang Local Chinese Medicine Research, School of Pharmaceutical Sciences, Hubei University of Medicine, Shiyan, Hubei, China

**Keywords:** cardiovascular disease, chronotherapy, melatonin receptor agonist, platelet circadian rhythm, REV-ERBα, thrombosis

## Abstract

Platelet function and count display robust circadian oscillations with a period of approximately 24 hours, governed by the core molecular clock and modulated by environmental factors such as light, diet, and stress. This review elaborates on three key aspects. First, platelet surface markers peak around 8–9 AM, whereas platelet count, ATP release, and aggregability reach their maximum between 3–8 PM. Mechanistically, the nuclear receptor Rev-erbα drives the morning surge in platelet reactivity and thrombosis via the OPHN-1/RhoA/ERM signaling pathway. Second, through their circadian rhythm, platelets actively participate in the pathogenesis of cardiovascular diseases, neurological disorders, and metabolic diseases. Third, circadian-targeting drugs including tasimelteon, ramelteon, agomelatine, SR9009, VTP-43742, and PF-670462 offer novel therapeutic strategies for rhythm-related disorders. This review highlights the circadian regulation of platelets as an emerging therapeutic target in human diseases.

## 1 Introduction

The circadian rhythm encompasses endogenous physiological and behavioral oscillations that occur spontaneously within an organism with a periodicity of approximately 24 hours. Its primary evolutionary purpose is to synchronize the organism’s internal homeostasis with external diurnal variations. Widely conserved from prokaryotes to higher mammals, this rhythmic regulation spans a multitude of life activities including the sleep-wake cycle, hormone secretion, metabolism, body temperature, and immunity serving as a cornerstone for physiological homeostasis (Panda et al., 2002; Takahashi, 2017; Hastings et al., 2018). At the molecular level, the circadian rhythm is driven by an intracellular transcription-translation feedback loop (TTFL), centered around core clock genes such as Clock, Bmal1, Per, and Cry, and their corresponding protein products (King et al., 1997; Bunger et al., 2000; Ko and Takahashi, 2006; Partch et al., 2014).

Mechanistically, CLOCK and BMAL1 proteins heterodimerize within the cytoplasm and translocate into the nucleus, where they bind to E-box elements in target gene promoters to initiate the transcription of Per (Per1/2/3) and Cry (Cry1/2) (Ko and Takahashi, 2006). Subsequently, translated PER and CRY proteins complex in the cytoplasm and retro-translocate into the nucleus, where they repress the transcriptional activity of the CLOCK/BMAL1 heterodimer, thereby forming a self-limiting negative feedback loop (Ko and Takahashi, 2006; Partch et al., 2014). This core loop is further stabilized and fine-tuned by nuclear receptors like REV-ERB and ROR, which modulate *Bmal1* expression (Preitner et al., 2002; Reppert and Weaver, 2002). Concurrently, the stability and degradation kinetics of PER and CRY proteins are post-translationally regulated via phosphorylation by kinases such as Casein Kinase 1 (CK1δ/ϵ), ensuring the precise 24-hour periodicity (Reppert and Weaver, 2002). To date, the circadian clock has been shown to dictate the expression of over 20% of the genome, thereby governing vital pathways such as energy metabolism, cell proliferation, DNA repair, immunity, and endocrine secretion. This complex network is hierarchically governed by the central pacemaker located in the suprachiasmatic nucleus (SCN) of the hypothalamus, which orchestrates peripheral tissue rhythms via neural and humoral pathways to maintain systemic alignment. A growing body of evidence indicates that circadian disruptions are heavily implicated in the pathogenesis of metabolic syndrome, malignancies, and neurodegenerative diseases (Hastings et al., 2018).

Platelets, small, anucleate blood cells derived from bone marrow megakaryocytes are classically recognized for their indispensable roles in hemostasis and thrombosis. However, emerging paradigms have expanded their functional repertoire, underscoring their involvement in diverse physiological and pathological processes, including immunity, inflammation, and tumor metastasis. During vascular injury, platelets adhere to the exposed subendothelial matrix, undergo activation, and aggregate to form a primary hemostatic plug. Simultaneously, they provide a procoagulant surface that accelerates thrombin generation and stabilizes the definitive clot. Through these mechanisms, platelets drive all three distinct waves of hemostasis initial platelet plug formation, the subsequent coagulation cascade, and the recently characterized “protein wave” marked by plasma fibronectin deposition (Muller et al., 1989). In addition to their hemostatic functions, platelets serve as potent immune modulators capable of recognizing, sequestering, and neutralizing pathogens. By recruiting and activating leukocytes, they enhance phagocytosis and trigger the release of neutrophil extracellular traps (NETs) (Tofler et al., 1987). This multifaceted interaction among platelets, the endothelium, leukocytes, and coagulation factors coordinates the process of thromb oinflammation, which is further amplified by the platelet release of growth factors, chemokines, and extracellular vesicles (Franco et al., 2015). Conversely, thrombocytopenia driven by accelerated clearance or impaired productionpredisposes patients to severe hemorrhage, posing life-threatening risks in disorders like immune thrombocytopenic purpura. In the context of cardiovascular pathology, the primary clinical impact of platelets lies in their capacity to physically occlude vessels through massive aggregation during acute ischemic events. While their ability to scaffold coagulation factors and promote the clotting cascade is well-established, this coagulation-centric mechanism remains secondary to aggregation-driven mechanical obstruction in coronary artery occlusion (Jackson, 2011). Uncontrolled platelet aggregation can culminate in abrupt vessel occlusion, triggering myocardial infarction or ischemic stroke. Furthermore, platelets actively contribute to deep vein thrombosis, thromboembolism, autoimmune diseases, and chronic inflammatory states by interacting with immune cells and sustaining the localized inflammatory milieu (Koupenova et al., 2018).

## 2 The phenomenon of circadian rhythm in platelet

**Figure 1:**
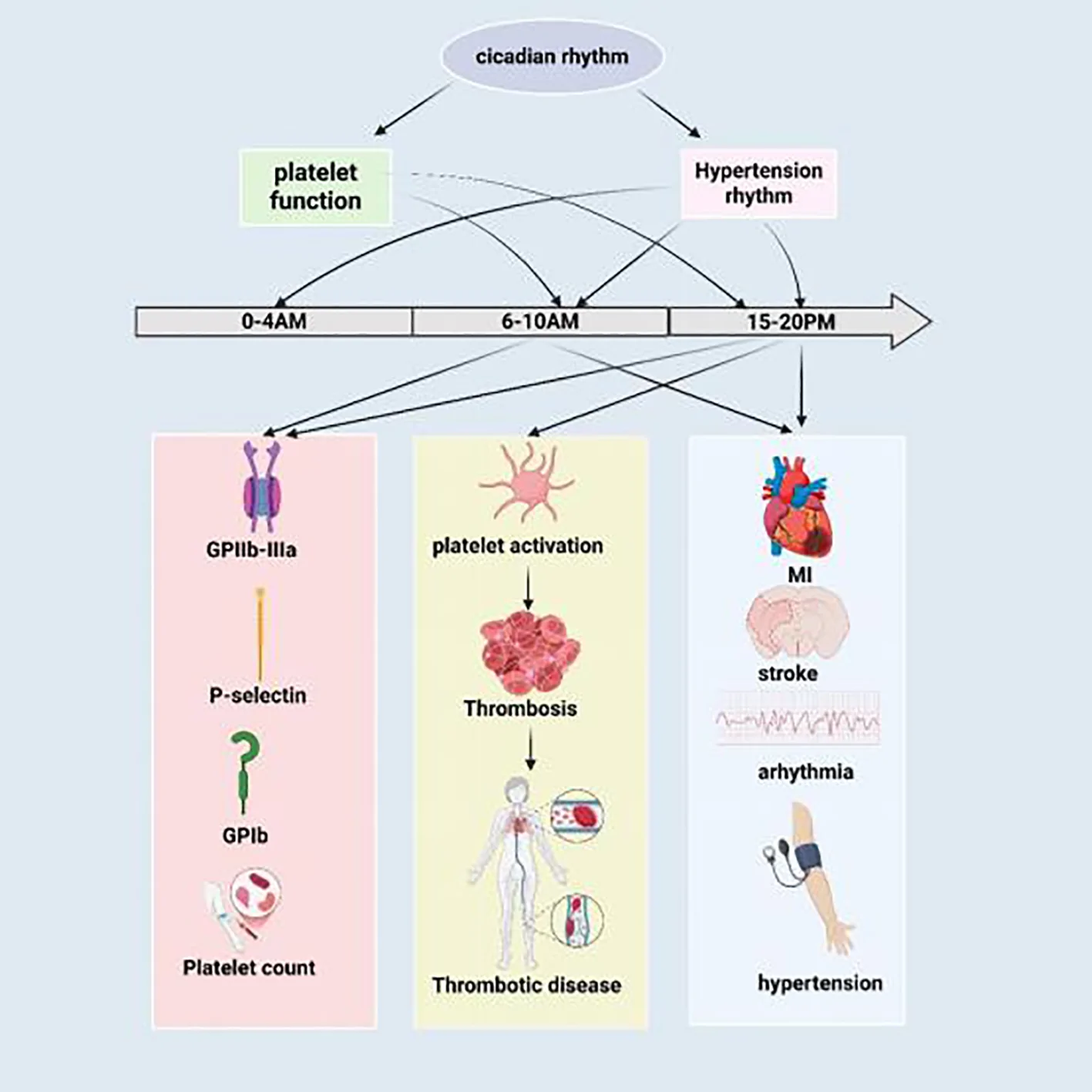
The phenomenon of circadian rhythm in platelet. This figure illustrates the influence of the circadian rhythm on platelet function and hypertension. Several key time periods are marked on the graph (such as 0–4 AM, 6–10 AM, 15–20 PM), and multiple related biomarkers and diseases are listed, including platelet-related indicators: GPlib-IIia, P-selectin, GPlb, platelet count, platelet activation, thrombosis and diseases, thrombosis formation, thrombotic diseases, myocardial infarction, stroke, cardiovascular rhythm abnormalities, arrhythmia, hypertension. These contents suggest that platelet activity and the occurrence of cardiovascular events may be regulated by the circadian rhythm, especially during the morning period (6–10 AM), with a higher risk.

Research has established that adverse cardiovascular events, such as myocardial infarction and stroke, exhibit a peak incidence in the morning (Muller et al., 1989). This morning surge is intimately linked to the endogenous circadian system, which drives daily oscillations in platelet activation and aggregability (Tofler et al., 1987). As critical components of the hemostatic and vascular systems, platelets exhibit distinct diurnal variations that prime the cardiovascular system for embolic events. Specifically, key platelet surface markers such as GPIIb-IIIa, GPIb, and P-selectin elevate significantly during the biological morning, peaking around 8:00–9:00 AM (Guillaumond et al., 2005; Leone et al., 2015). Interestingly, while early-morning activation facilitates initial thrombus formation, other parameters, including total platelet count, ATP release, and maximal aggregability, display a distinct circadian phase that peaks later in the afternoon and evening (3:00–8:00 PM). This temporal regulation of platelet activity directly aligns with the clinical epidemiology of thrombosis-related diseases, whose incidence and mortality have risen sharply in recent years. Beyond platelet dynamics, the endogenous biological clock orchestrates a broader synchronization of the cardiovascular system, leading to a cluster of acute events during the morning hours. For instance, arrhythmias, sudden cardiac death, and acute myocardial infarction manifest predominantly at this time (Portaluppi et al., 2012). A classic parallel is observed in the diurnal blood pressure profile of hypertensive patients, which classically exhibits a two-peak, one-trough pattern (Hermida et al., 2007). This rhythm is characterized by a primary morning surge between 06:00 and 10:00, a secondary afternoon peak from 16:00 to 18:00, and a nocturnal nadir between 00:00 and 04:00 (Millar-Craig et al., 1978). Together, these synchronized circadian fluctuations create a high-risk physiological window in the morning, accelerating thrombosis and precipitating acute embolic disorders (**Figure 1**).

## 3 The multi-layered mechanism driving platelet circadian rhythmicity

**Figure 2:**
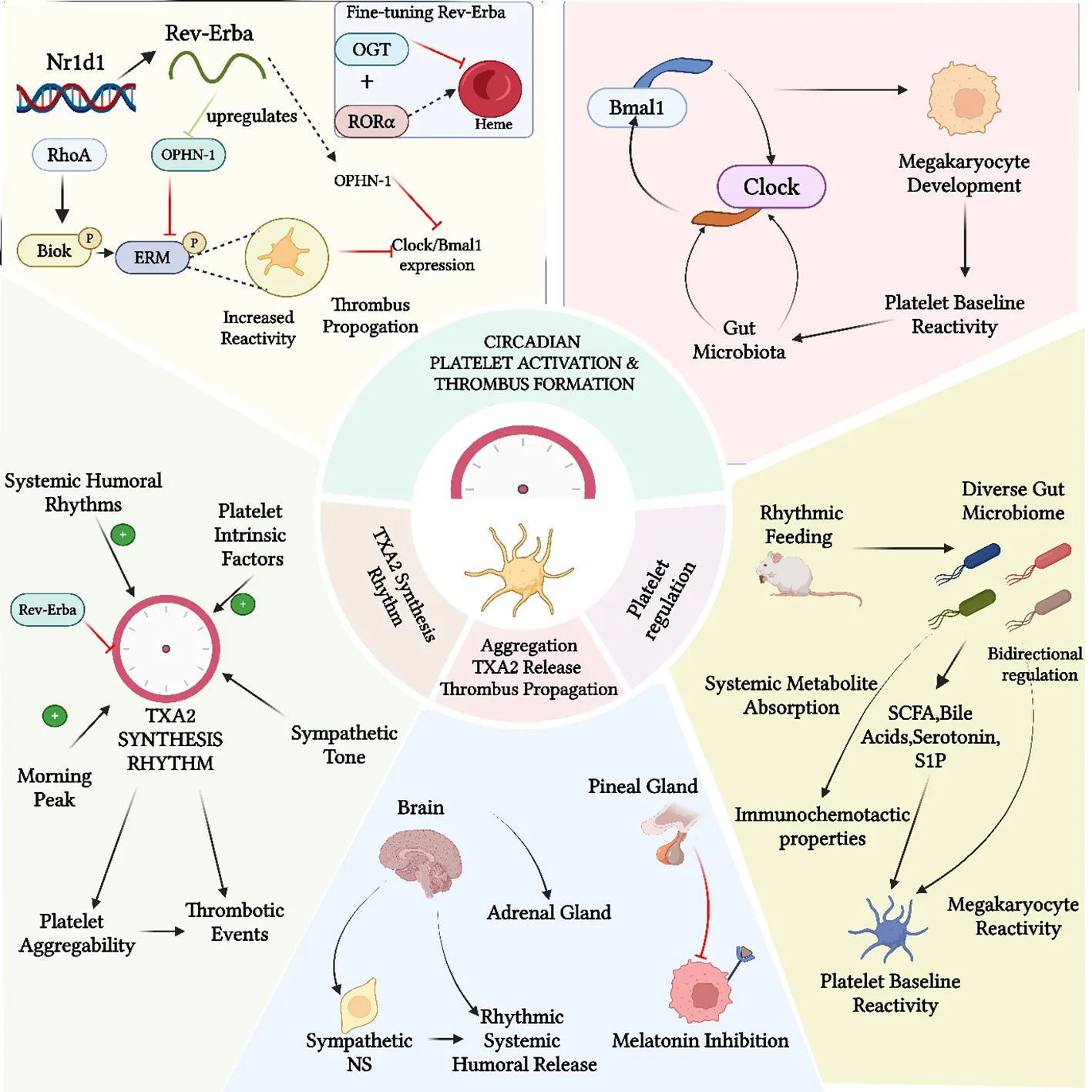
Integrative regulatory network of circadian platelet rhythms and atherothrombotic risk. This schematic diagram illustrates the multi-layered molecular, neuroendocrine, and metabolic pathways that govern diurnal variations in platelet reactivity, culminating in the characteristic morning surge of thrombotic events. Central to this network is the rhythmic activation of circulating platelets and thrombus formation (center). Mechanistically, the Nr1d1-encoded circadian nuclear receptor Rev-Erbα acts as a pivotal positive driver of platelet activation; it exhibits robust circadian oscillations and upregulates platelet reactivity and thrombus propagation via the OPHN-1-mediated RhoA/ERM signaling pathway (top left). The stability and transcriptional activity of Rev-Erbα are further fine-tuned by a regulatory loop involving O-GlcNAc transferase (OGT), RORα, and intracellular heme, while downstream, Rev-Erbα and Rev-Erbβ feedback to repress core clock genes Clock and Bmal1. Concurrently, core transcription-translation feedback loop (TTFL) components (Bmal1 and Clock) intrinsically regulate megakaryocyte development and thrombopoiesis, thereby dictating baseline circulating platelet reactivity (top right). External systemic inputs further modulate this intrinsic clockwork (bottom panels): the hypothalamic-pituitary-adrenal (HPA) axis and sympathetic nervous system drive the rhythmic release of cortisol and catecholamines to stimulate systemic humoral rhythms, whereas pineal melatonin acts through surface receptors to inhibit platelet activation. Parallelly, host feeding rhythms shape the diversity of the gut microbiota, driving the cyclical production and systemic absorption of microbial metabolites such as short-chain fatty acids (SCFAs), secondary bile acids, serotonin (5-HT), and sphingosine 1-phosphate (S1P), which exert bidirectional epigenetic and transcriptional control over peripheral clock genes and immunochemotactic properties. Ultimately, these pathways intersect with a pronounced daily rhythm in thromboxane A2 TXA2) biosynthesis. Driven by sympathetic tone and cortisol fluctuations, this TXA2 peak enhances platelet aggregability independently of baseline clock transcription, creating a high-risk morning window for acute cardiovascular events and highlighting potential targets for personalized chronotherapy.

Growing evidence originally positioned the circadian nuclear receptor Rev-Erbα (encoded by Nr1d1) as a pivotal driver of platelet rhythmicity (**Figure 2**). Rev-Erbα exhibits robust circadian oscillations in human platelets, driving the morning surge in thrombotic risk by upgrading platelet reactivity via the oligophrenin-1 (OPHN-1)-mediated RhoA/ERM signaling pathway (Zhao et al., 2011; Shi et al., 2022). However, an exclusive reliance on the Rev-Erbα paradigm oversimplifies platelet chronobiology and neglects a critical architectural paradox: as anucleate cells, circulating platelets lack active nuclear transcription-translation feedback loops (TTFLs) (Thaiss et al., 2014; Liang et al., 2015). Consequently, alternative cell-autonomous mechanisms and external humoral/neuroendocrine oscillations must be evaluated to understand this temporal meshwork. Intrinsically, core clock components such as BMAL1 and CLOCK operate primarily during the megakaryocyte differentiation phase in the bone marrow, imposing a transcriptionally “pre-programmed” baseline of reactivity and lifespan onto newly shed circulating platelets (Von Kanel et al., 2004; Somanath et al., 2011; Hearn et al., 2023). Beyond this inherited clockwork, circulating platelets harbor translation-independent, non-transcriptional metabolic oscillators, typified by the 24-hour rhythmic hyperoxidation of peroxiredoxins, which orchestrates diurnal energy states and redox-dependent signaling (Pagani et al., 2010; Vandenberghe et al., 2022).

Systemically, the hypothalamic-pituitary-adrenal (HPA) axis and the sympathetic nervous system impose strong circadian control via the rhythmic release of cortisol and catecholamines (King et al., 1997; Bunger et al., 2000). Concurrently, pineal melatonin serves as a vital systemic brake, binding to platelet MT1/MT2 receptors to blunt activation during the biological night (Muller et al., 1989; Preitner et al., 2002). Crucially, this neuroendocrine network couples with a pronounced daily rhythm in the biosynthesis of thromboxane A2 (TXA2), a potent pro-aggregatory and vasoconstrictive eicosanoid (Tofler et al., 1987; Hermida et al., 2005). The excretion of major TXA2 metabolites (e.g., urinary 11-dehydro-TXB2) peaks sharply in the morning hours (Tofler et al., 1987; Hermida et al., 2007). This diurnal variation in TXA2 production is tightly governed by systemic sympathetic tone and cortisol fluctuations, directly magnifying the baseline aggregation capacity of platelets independently of immediate gene transcription (Tofler et al., 1987; King et al., 1997).

## 4 Platelet regulates human diseases through circadian rhythm

### 4.1 Platelet regulates cardiovascular diseases through circadian rhythm

Acute myocardial infarction (AMI) exhibits a striking temporal clustering, with incidence skyrocketing between 06:00 AM and 12:00 PM (Panda, 2016; Hastings et al., 2018). In the context of coronary artery pathology, the primary clinical impact of platelets lies in their capacity to physically occlude vessels through massive aggregation during acute ischemic events (Jackson, 2011). While their ability to scaffold coagulation factors and promote the clotting cascade is well-established, this coagulation-centric mechanism remains secondary to aggregation-driven mechanical obstruction in coronary artery occlusion (Jackson, 2011; Budkowska et al., 2019). Uncontrolled platelet aggregation at the site of a ruptured atheromatous plaque culminates in abrupt vessel occlusion, triggering downstream myocardial necrosis (Portaluppi et al., 2012; Budkowska et al., 2019).

This high-risk morning window is driven by a physiological confluence: the early morning zenith of Rev-Erbα expression (at 09:00 AM) triggers inside-out activation of integrin αIIbβ3 and α-granule exocytosis (Shi et al., 2022), while a synchronous surge in endogenousTXA2 biosynthesis amplifies recruitment and aggregation dynamics (Tofler et al., 1987; Hermida et al., 2005).

**Figure 3:**
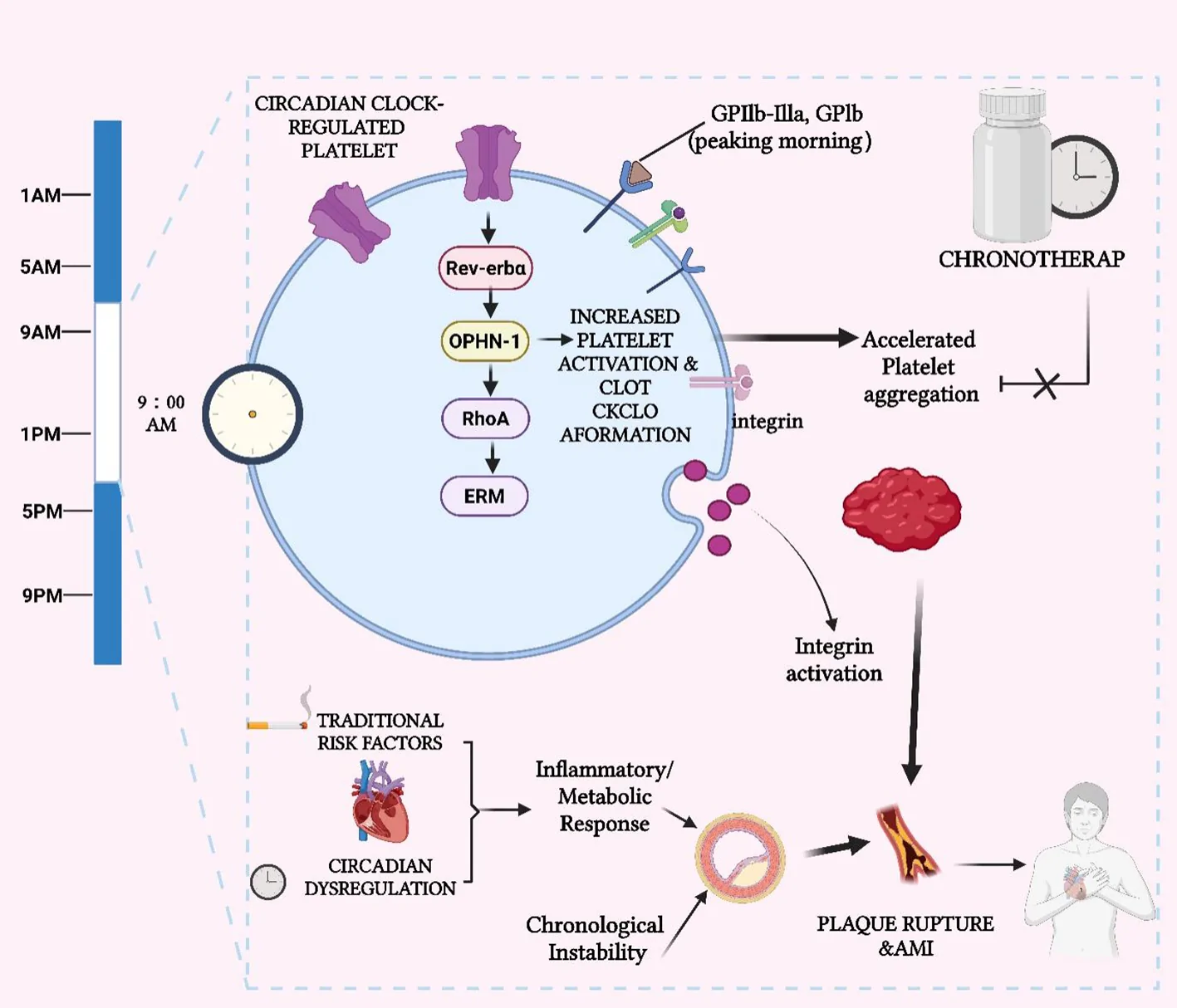
Circadian clock-regulated platelet reactivity and its role in morning acute myocardial infarction (AMI) pathogenesis and chronotherapeutic intervention. The bimodal diurnal rhythm significantly influences the transition from chronic stable coronary artery disease (CAD) to acute cardiovascular events. In the biological morning (around 09:00 AM), a physiological high-risk window opens, driven by traditional risk factors (e.g., smoking, sedentary lifestyle, pollution, and infections) and endogenous circadian dysregulation that induce inflammatory and metabolic instabilities to establish the foundational pathological substrate. At the molecular level, this temporal clustering of AMI is catalyzed by cell-autonomous clock receptors within platelets, notably Rev-Erbα, which peaks synchronously at approximately 09:00 AM to potentiate activation and thrombus propagation. Mechanistically, Rev-Erbα serves as a critical positive regulator of platelet reactivity by activating the oligophrenin-1 (OPHN-1)-mediated RhoA/ERM (ezrin/radixin/moesin) signaling cascade, which triggers integrin inside-out activation and α-granule exocytosis. This intracellular cascade synergizes with the endogenous morning spike of vital surface markers such as GPIIb-IIIa and GPIb, leading to dramatically increased platelet activation, clot formation, and accelerated platelet aggregation. When an unstable atherosclerotic plaque ruptures under these morning hemodynamic surges, these highly reactive platelets orchestrate sustained mechanical occlusion, culminating in catastrophic ischemic events (AMI). Conversely, strategic chronotherapy such as time-sensitive drug administration or pharmacological Rev-Erbα inhibition can blunt this critical morning spike in platelet reactivity, thereby maximizing therapeutic efficacy and mitigating acute cardiovascular risks.

To contextualize these findings, the clinical impact of these circadian variations must be compared against traditional cardiovascular risk factors, including smoking, lifestyle, pollution, and microbial infections (Bowman et al., 2018; Maki and Duell, 2024). While traditional risk factors establish the foundational pathological substrate such as structural plaque progression over decades (Maki and Duell, 2024), circadian misalignment and intrinsic clock dysregulation function as independent, acute temporal catalysts (Panda, 2016; Shi et al., 2022). Rather than operating in isolation, these systems act synergistically: traditional factors create the unstable structural architecture, whereas intrinsic circadian fluctuations in hemodynamics and platelet aggregability dictate the precise temporal window of plaque rupture and mechanical occlusion (Jackson, 2011; Hastings et al., 2018; Shi et al., 2022) (**Figure 3**).

### 4.2 Platelet regulates nervous system diseases through circadian rhythm

Neurological diseases encompass a broad spectrum of disorders affecting the central nervous system (the brain and spinal cord) and the peripheral nervous system, including peripheral nerves, nerve roots, neuromuscular junctions, and muscles. According to the Global Burden of Disease (GBD) 2021 study, approximately 3.7 billion people worldwide accounting for 43.1% of the global population are affected by neurological conditions, rendering them the leading contributor to the global disease burden with an aggregate of 443 million disability-adjusted life years (DALYs) (Collaborators G B D N S D, 2024). Stroke remains the primary driver of both mortality and DALYs, followed closely by neonatal encephalopathy, Alzheimer’s disease (AD), and migraine (Feigin et al., 2022; Collaborators G B D N S D, 2024). Paradoxically, while age-standardized mortality and DALY rates declined between 1990 and 2021 due to medical advancements, absolute DALYs escalated by 18.2%, reflecting an expanding total burden driven primarily by population aging (Feigin et al., 2022; Collaborators G B D N S D, 2024). Key risk factors, such as elevated systolic blood pressure, low birth weight, preterm birth, and metabolic syndrome, exert profound impacts, particularly on stroke incidence (Feigin et al., 2022). Notably, the neurological disease spectrum exhibits stark regional disparities infectious neurological diseases predominate in low-income nations, whereas non-communicable neurodegenerative disorders dominate high-income economies (Collaborators G B D N S D, 2024). Because most of these progressive degenerative conditions currently lack effective, modifiable risk factors, innovative preventative and therapeutic strategies are urgently required.

Emerging paradigms indicate that the circadian rhythm of platelets plays a crucial role in maintaining nervous system homeostasis (Musiek and Holtzman, 2016). Beyond their classical role in hemostasis, platelets serve as potent inflammatory modulators and peripheral biomarkers in neurological diseases, including AD and amyotrophic lateral sclerosis (ALS) (Leiter and Walker, 2020). Because platelets and neurons share remarkable structural and biochemical characteristics such as homologous secretory granules and shared surface receptors dynamic alterations in platelet reactivity effectively mirror neuronal injury and disease progression, offering a minimally invasive window for early diagnosis and therapeutic monitoring (Fleury et al., 2021). Platelet function is strictly regulated by the endogenous circadian clock, exhibiting distinct diurnal fluctuations in activation, metabolism, and vesicular secretion (Scheer et al., 2011; Fonken et al., 2015). Crucially, the platelet mitochondrial melatonin pathway interlocks with these circadian networks, modulating thromboinflammatory cascades and shaping the neural microenvironment (Sweeney et al., 2018; Anderson et al., 2019). Concurrently, platelet-derived bioactive molecules, such as serotonin (5-HT) and sphingosine 1-phosphate (S1P), cross-talk with the gut-brain axis, direct immune cell chemotaxis, and modulate glial cell activity, thereby indirectly orchestrating CNS pathophysiological processes (Rust et al., 2023).

At the molecular level, the tripartite interplay among circadian rhythms, platelet dynamics, and neurological diseases converges on core clock networks, neuroendocrine signals, and cellular quality control pathways. The autonomous transcription-translation feedback loops (TTFLs) driven by core clock genes including Clock, Bmal1, Per, and Cry dictate baseline cellular metabolism, redox balance, mitochondrial homeostasis, and synaptic plasticity within both central neurons and circulating platelets (Hood and Amir, 2017). Melatonin signaling further modulates this network by binding to specific platelet and neural receptors to suppress chronic neuroinflammation (Sweeney et al., 2018). Chronobiological disruption profoundly destabilizes this delicate homeostatic network through several distinct mechanisms.

**Figure 4:**
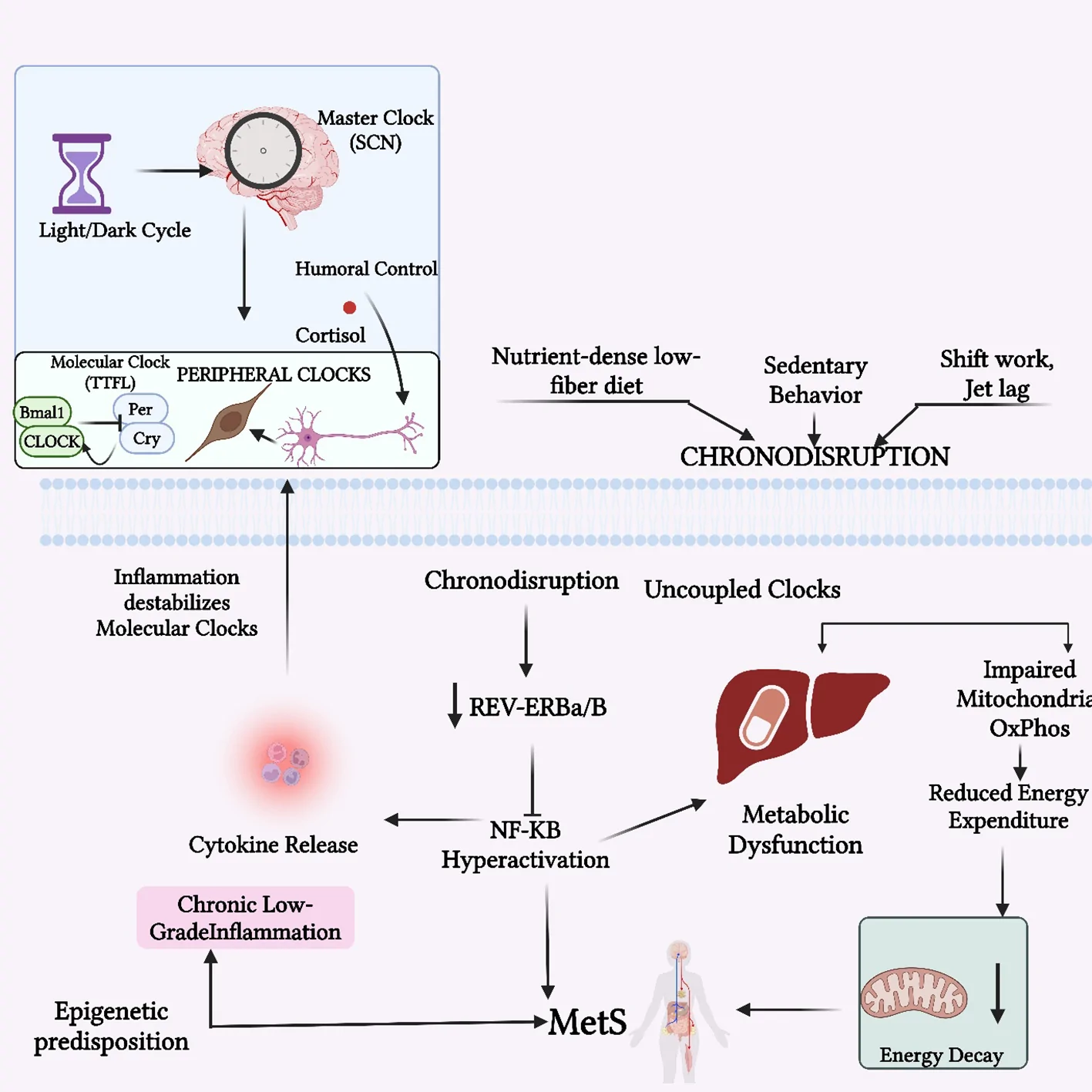
The bidirectional pathogenic feedback loop between chronodisruption and metabolic syndrome (MetS). Under physiological conditions, circadian homeostasis is hierarchically regulated by the master pacemaker in the suprachiasmatic nucleus (SCN), which responds to environmental light/dark cycles and synchronizes peripheral clocks (in the liver, white adipose tissue, and skeletal muscle) via humoral control, such as the diurnal oscillation of cortisol. At the cellular level, these peripheral clocks are driven by an autonomous transcription-translation feedback loop (TTFL) consisting of core clock genes (Bmal1, Clock, Per, and Cry). However, modern environmental stressors including nutrient-dense, low-fiber diets, sedentary behavior, shift work, and jet lag induce chronodisruption, which uncouples peripheral oscillators from the central pacemaker. This circadian misalignment downregulates the expression of nuclear receptors REV-ERBα/bβ, relaxing their transcriptional brake on pro-inflammatory pathways and leading to the hyperactivation of nuclear factor kappa B (NF-κB). Consequently, increased cytokine release (e.g., IL-6, CCL2) establishes a state of chronic low-grade inflammation, which feeds back to further destabilize peripheral molecular clocks. Simultaneously, uncoupled peripheral clocks directly cause metabolic dysfunction, manifesting as insulin resistance, dyslipidemia, and hepatic steatosis, while also impairing mitochondrial oxidative phosphorylation (OxPhos). This impairment reduces overall energy expenditure, precipitating systemic energy decay. Intersected with epigenetic predispositions, this self-sustaining, bidirectional loop among chronodisruption, hyperinflammation, and metabolic decay ultimately culminates in and exacerbates the clinical phenotype of Metabolic Syndrome (MetS), characterized by central obesity, insulin resistance, hypertension, dyslipidemia, and hyperinflammation.

First, circadian misalignment impairs proteostasis, accelerating the cytotoxic aggregation of misfolded proteins, such as amyloid-βAβ and hyperphosphorylated tau, which hallmarks neurodegenerative pathogenesis (Morrone et al., 2023). Second, the molecular clock directly gates the transcription of pro-inflammatory cytokines (e.g., IL-6 and TNFα); its dysregulation consequently unleashes unchecked neuroinflammation and exacerbates neuronal apoptosis (Fonken et al., 2015). Third, core clock networks dictate the circadian expression of key tight junction proteins and efflux transporters within the blood-brain barrier (BBB), modulating its structural permeability (Liao et al., 2025). Clock gene dysfunction disrupts this endothelial barrier, allowing systemic neurotoxins and inflammatory cells to infiltrate the CNS parenchyma (Liao et al., 2025). Concurrently, the collapse of clock-controlled antioxidant systems triggers severe oxidative stress and mitochondrial bioenergetic failures, accelerating neurodegenerative decay (Namgyal and Lim, 2025). In summary, the breakdown of these interlocked pathways spanning core molecular clocks, melatonin signaling, platelet function, and BBB integrity jointly drives the pathogenesis of neurological disorders, defining a promising frontier for chronotherapeutic interventions (**Figure 4**).

### 4.3 Platelet regulates metabolic diseases through circadian rhythm

Metabolic Syndrome (MetS) clusters a constellation of metabolic abnormalities, including central obesity, insulin resistance, systemic hypertension, and dyslipidemia. Driven by the global shift toward westernized lifestyles and demographic aging, the prevalence of MetS has escalated dramatically, positioning it as a primary driver of type 2 diabetes mellitus (T2DM) and cardiovascular diseases (Noubiap et al., 2025). At the chronic systemic level, low-grade inflammation and oxidative stress constitute the core pathogenic triggers (Engin, 2017). Excess adiposity and high-calorie diets prompt adipose tissue to hypersecrete pro-inflammatory adipokines, which permanently activate inflammatory cascades and induce cellular damage, thereby worsening insulin resistance and endothelial dysfunction (Engin, 2017). Structurally, MetS possesses a highly heritable architecture involving hundreds of genetic loci, further shaped by epigenetic modifications such as altered DNA methylation profiles and aberrant miRNA expression (Fahed et al., 2022). These genetic predispositions intersect with environmental catalysts, including nutrient-dense, low-fiber diets and sedentary behavior, creating a multi-systemic pathological network that demands comprehensive lifestyle, pharmacological, and innovative chronotherapeutic interventions.

The maintenance of global metabolic homeostasis is hierarchically governed by the host circadian system, an endogenous 24-hour pacemaking network (Panda, 2016). This system coordinates nutrient utilization, endocrine secretion, and inflammatory profiles through an intracellular transcription-translation feedback loop (TTFL) driven by core clock genes, including Clock, Bmal1, Per, and Cry (Panda, 2016). Circadian alignment is managed via an intricate synchronization between the master pacemaker in the suprachiasmatic nucleus (SCN) of the hypothalamus and autonomous peripheral clocks situated in metabolic tissues, such as the liver, white adipose tissue, and skeletal muscle (Bass and Lazar, 2016). The SCN orchestrates these peripheral oscillators through neural and humoral conduits notably the hypothalamic-pituitary-adrenal (HPA) axis driving the diurnal oscillation of glucocorticoids (e.g., cortisol) to fine-tune cellular stress responses and energetic efficiency (Oster et al., 2017). At the pathway level, the molecular clock gates insulin sensitivity, glucose transporter expression, and fatty acid oxidation kinetics (Poggiogalle et al., 2018).

**Figure 5:**
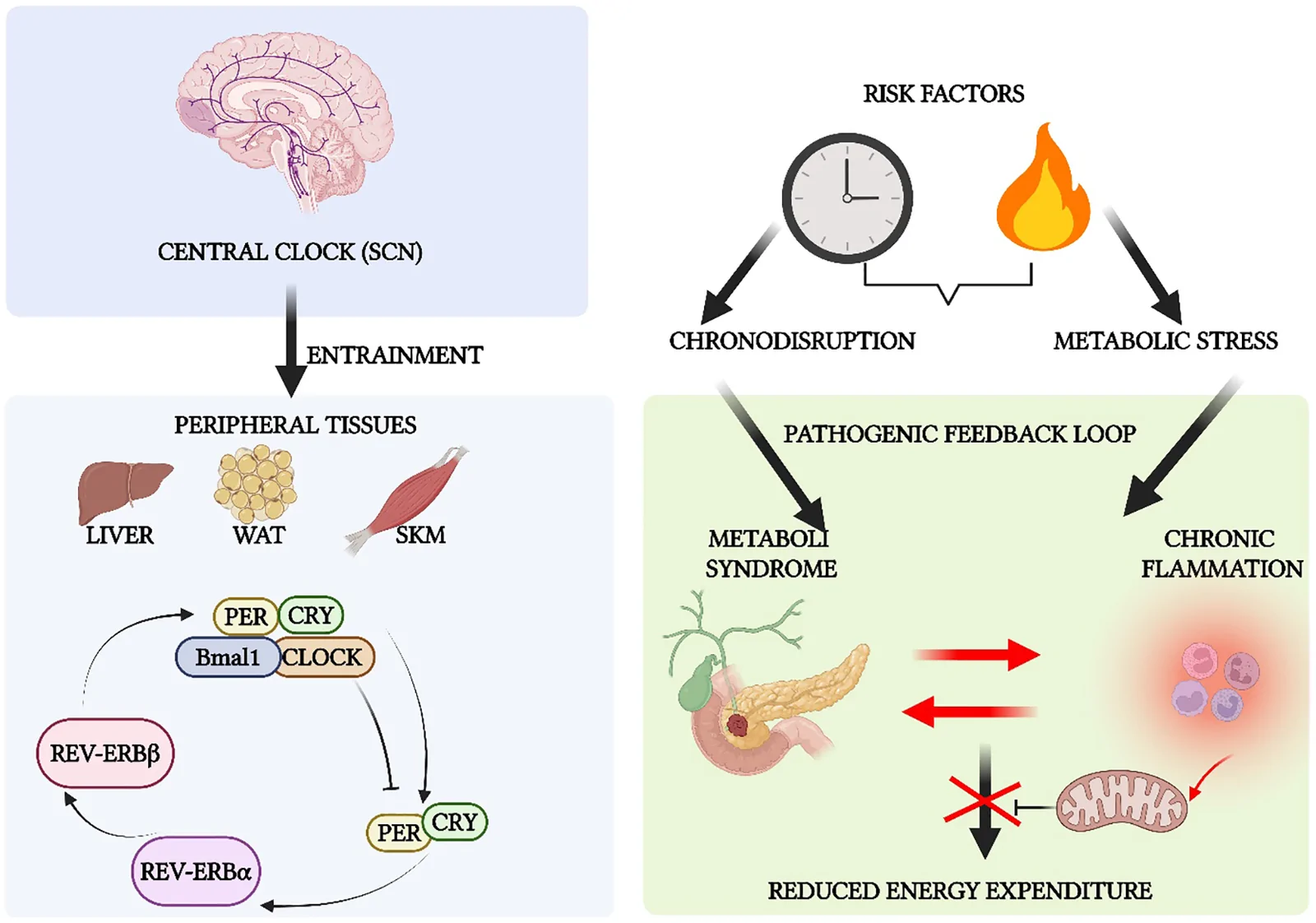
Schematic diagram illustrating the crosstalk between the central circadian clock and peripheral metabolic tissues, and the consequences of chronodisruption on metabolic health. The central clock located in the suprachiasmatic nucleus (SCN) is entrained by external light-dark cycles (entrainment), and subsequently coordinates peripheral oscillators in key metabolic organs including the liver, white adipose tissue (WAT), and skeletal muscle (SKM). Within these peripheral tissues, a core transcriptional–translational feedback loop is formed by the positive regulators CLOCK and BMAL1, which drive the expression of the negative regulators PER and CRY, as well as the nuclear receptor REV-ERB. REV-ERB in turn represses BMAL1 transcription, establishing a robust self-sustained circadian rhythm. Under physiological conditions, this system ensures temporal orchestration of metabolism. However, when chronodisruption (circadian disruption) occurs due to genetic, environmental, or behavioral risk factors it imposes metabolic stress on peripheral tissues. Such stress triggers a pathogenic feedback loop characterized by reduced energy expenditure and chronic low-grade inflammation. The convergence of these pathological events ultimately promotes the development of metabolic syndrome, highlighting the critical role of circadian integrity in maintaining systemic metabolic homeostasis.

Consequently, chronodisruption induced by modern environmental stressors like shift work, jet lag, and irregular feeding schedules uncouples these peripheral clocks from the central pacemaker, precipitating insulin resistance, hepatic steatosis, and profound dyslipidemia (Maury et al., 2010). Beyond direct metabolic gating, molecular clock proteins interlock with innate immune pathways to control chronic inflammation. For instance, the clock-controlled nuclear receptors REV-ERBα/β directly repress the transcription of pro-inflammatory cytokines, including IL-6 and CCL2 (Sato et al., 2014). Circadian disruption relaxes this transcriptional brake, triggering the hyperactivation of the NF-κB pathway and establishing a state of chronic, low-grade metabolic inflammation (Green et al., 2008). Crucially, this relationship is reciprocal metabolic stress and adiposity-driven inflammation feed back to destabilize peripheral molecular clocks (Kohsaka et al., 2007). This bidirectional uncoupling forms a pathogenic feedback loop inflammation, circadian misalignment, and metabolic decay which significantly impairs mitochondrial oxidative phosphorylationn and reduces overall energy expenditure, further exacerbating the MetS phenotype (Poggiogalle et al., 2018) (**Figure 5**).

### 4.4 The circadian rhythm regulates intestinal microorganisms

Furthermore, the bidirectional cross-talk between host circadian rhythms and the gut microbiome has emerged as a critical driver of systemic metabolic, cardiovascular, and neurological disorders (Bautista and Lopez-Cortes, 2025). Host behavioral rhythms, particularly feeding-fasting cycles, strictly entrain the diurnal fluctuations and taxonomic composition of the commensal microflora (Bautista and Lopez-Cortes, 2025; Bautista et al., 2025a). Conversely, microbial communities actively modulate host peripheral clocks by generating bioactive metabolites, such as short-chain fatty acids (SCFAs; e.g., butyrate) and trimethylamine N-oxide (TMAO), which serve as systemic signaling inputs to regulate peripheral *Bmal1* and *Clock* expression (Fawad et al., 2022; Dos Santos and Vasylyshyn, 2025). When triggered by shift work or erratic eating, the breakdown of this host-microbiome clock axis drives profound gut dysbiosis and compromises intestinal barrier integrity, leading to metabolic endotoxemia (Thaiss et al., 2014; Grasa-Ciria et al., 2025). In metabolic disorders, this endotoxemia chronically alters clock-gene expression in adipose and hepatic tissues, accelerating obesity and insulin resistance (Frazier et al., 2020).

Strikingly, this sustained metabolic inflammation primes circulating platelets for systemic hyperreactivity (Kruger-Genge et al., 2020). In cardiovascular pathways, dysbiosis-altered metabolites, such as elevated systemic TMAO, directly accelerate platelet activation and aggregation, exacerbating the morning surge in thrombotic risk governed by the host’s misaligned biological clock. In neurological conditions, the collapse of microbiome-host clock synchrony disrupts the gut-brain axis, promoting neuroinflammation and blood-brain barrier dysfunction via aberrant platelet-glial interactions (Rust et al., 2023). Recognizing and maintaining the circadian synergy within this host-microbiome-platelet axis is therefore vital to preventing systemic thromboinflammatory cascades across multi-organ disease spectrums (Bautista et al., 2025a).

The host circadian system tightly orchestrates the diurnal composition and biogeographical distribution of the gut microbiota via rhythmic feeding-fasting cycles (Bonten et al., 2014; Ma et al., 2023). Conversely, microbial-derived metabolites absorbed into the systemic circulation (e.g., short-chain fatty acids SCFAs and secondary bile acids) exert epigenetic and transcriptional control over peripheral clock genes (Bmal1, Per, Cry) in peripheral tissues (Ma et al., 2023), potentially targeting bone marrow megakaryocytes (Kim et al., 2013).

Under chronodisruption, this host-microbiome clock axis collapses, inducing gut dysbiosis and compromising intestinal barrier integrity, leading to metabolic endotoxemia (Bonten et al., 2014). In cardiovascular pathways, dysbiosis-altered metabolites, such as elevated systemic trimethylamine N-oxide (TMAO), directly accelerate platelet activation and aggregation, exacerbating the morning surge in thrombotic risk governed by the host’s misaligned biological clock (Bonten et al., 2014; Ma et al., 2023).

### 4.5 Clinical evidence of the circadian influence on antiplatelet therapy

The profound impact of the circadian rhythm on platelet activation naturally extends to the clinical efficacy of antiplatelet agents. Traditional administration of antiplatelet therapies has largely ignored the time-of-day variation; however, chronotherapy has revealed that the timing of drug intake significantly alters patient outcomes (Buurma et al., 2019). Given aspirin’s short half-life, administering it at bedtime rather than in the morning could significantly improve platelet inhibition during the crucial early morning hours, which are associated with increased cardiovascular risk (Nadeem et al., 2024). From a mechanistic perspective, the body’s intrinsic circadian rhythm ensures that aspirin’s anti-aggregatory effects are maximized when new platelets are primarily released overnight (Shi et al., 2022). Furthermore, the expression of the circadian nuclear receptor Rev-erbα in platelets has been directly linked to the morning peak in thrombotic events, suggesting that the timing of existing antiplatelet agents could be optimized by targeting this circadian window (Tual-Chalot and Stellos, 2022).

Similarly, research demonstrates that administering clopidogrel in the evening results in more potent and sustained platelet inhibition during the critical early-morning hours, as opposed to administration in the morning (Ma et al., 2023). The CLOCK genes modulate the diurnal rhythmicity of clopidogrel efficacy and toxicity by regulating the expression of key metabolic enzymes, including CYP1A2, CYP3A11, and CES1D (Ma et al., 2023). These findings may help optimize clopidogrel dosing regimens to precisely align with circadian metabolic peaks. In addition to direct antiplatelet effects, it is important to report that aspirin may regulate gut microbiota and influence host circadian mechanisms involved in inflammation and cardiovascular health (Ma et al., 2023). This bidirectional relationship suggests that aspirin’s cardioprotective properties are partially mediated through circadian-microbiome axes, further underscoring the importance of optimal administration timing (Ma et al., 2023). Future clinical strategies must comprehensively account for these circadian fluctuations to maximize the protective effects of drugs, ensuring peak plasma concentrations coincide with the biological morning surge in platelet aggregability (Bonten et al., 2014).

Reflecting these circadian rhythms, standard antiplatelet therapy demonstrates significant time-of-day variations in clinical efficacy (Tofler et al., 1987; Vandenberghe et al., 2022). Given aspirin’s short circulating half-life (20 minutes), traditional morning administration leaves a crucial vulnerability window (Tofler et al., 1987). Mechanistically, the body’s intrinsic circadian rhythm ensures that a substantial fraction of new platelets are primarily released overnight from the bone marrow (Von Kanel et al., 2004; Hearn et al., 2023). Morning dosing allows these uninhibited, highly reactive fresh platelets to accumulate throughout the day and night, leaving the patient under-protected during the high-risk early morning hours (Tofler et al., 1987). In contrast, bedtime administration of low-dose aspirin ensures that the peak drug availability aligns with overnight platelet release, effectively shifting the pharmacokinetics to maximize anti-aggregatory effects and blunt the morning spike in TXA2 synthesis (Tofler et al., 1987) (Hermida et al., 2005).

Similarly, research demonstrates that administering clopidogrel in the evening results in more potent and sustained platelet inhibition during the critical early-morning hours, as opposed to morning dosing (Vandenberghe et al., 2022). Host CLOCK genes modulate this diurnal rhythmicity of clopidogrel efficacy and toxicity by directly regulating the hepatic transcriptional expression of key metabolic activating enzymes (including CYP1A2 and CYP3A11) and inactivation hydrolases (CES1D) (Pagani et al., 2010; Vandenberghe et al., 2022).

## 5 Targeting drug for circadian rhythm to platelet

### 5.1 Tasimelteon

Tasimelteon is a novel dual-receptor melatonin agonist developed specifically for regulating and reconstructing circadian rhythm disorders, primarily in completely blind populations (Dhillon and Clarke, 2014; Lavedan et al., 2015). It activates melatonin MT1 and MT2 receptors, particularly exhibiting a higher affinity for the MT2 receptor, which directly acts on the suprachiasmatic nucleus (SCN) to reset the body’s biological clock (Lockley et al., 2015). Clinically, Tasimelteon holds FDA and EMA approval specifically for non-24-hour sleep-wake disorders in totally blind individuals (Nishimon et al., 2019), with its efficacy supported by high-quality, randomized, double-blind Phase III clinical trials (SET and RESET studies) that demonstrated significant increases in circadian entrainment and improved sleep duration. Regarding safety and limitations, the drug is generally well-tolerated, with mild to moderate adverse reactions including headache, transiently elevated liver enzymes, and abnormal dream (Bonacci et al., 2015; Leger et al., 2015). However, its application in other circadian disorders remains exploratory, and long-term direct comparative studies with traditional melatonin are lacking. Translationally, while tasimelteon does not directly bind to platelets, its profound ability to resynchronize the central pacemaker offers a pathway to indirectly modulate the neuroendocrine signals driving the morning peak of platelet activation, presenting a systemic approach to mitigating morning cardiovascular risk in chronodisrupted patients (Muller et al., 1985; Scheer et al., 2011) (**Table 1**).

### 5.2 Ramelteon

Ramelteon is a highly selective MT1/MT2 melatonin receptor agonist that regulates the circadian rhythm by simulating endogenous melatonin without acting on GABA receptors (Kato et al., 2005; Mishima, 2012). As an FDA-approved medication, it possesses robust clinical evidence from extensive human and animal studies confirming its efficacy in advancing or delaying circadian phases, making it a standard treatment for various circadian rhythm sleep disorders (Miyamoto, 2009). Ramelteon offers a superior safety profile compared to traditional sedatives, notably lacking side effects such as addiction, cognitive impairment, withdrawal syndrome, or rebound insomnia (Pandi-Perumal et al., 2011). Despite these advantages, a critical limitation is its strict dose-dependent and time-window sensitivity; low doses are effective only when administered at specific circadian phases, whereas higher doses unexpectedly lose their phase-shifting efficacy (Richardson et al., 2008). Because platelets express melatonin receptors, Ramelteon holds significant translational relevance as a safe, non-bleeding-inducing therapeutic tool (Scheer et al., 2011). By aligning the administration timing with optimal phase delays, it could dampen circadian-misaligned inflammatory and platelet responses, particularly in patients vulnerable to thrombotic events during shift work (Saharov et al., 2025) (**Table 1**).

### 5.3 Agomelatine

Agomelatine possesses a unique dual-action mechanism as an MT1/MT2 receptor agonist and a 5-HT receptor antagonist (Levitan et al., 2012). Supported by robust clinical trials, it is clinically utilized as a novel antidepressant that effectively alleviates depressive symptoms while simultaneously resetting disrupted circadian rhythms and improving sleep latency (Kasper et al., 2010; Levitan et al., 2012). While its efficacy often surpasses traditional antidepressants, its clinical application is limited by the necessity for routine monitoring of liver function due to rare but potential hepatotoxicity risks (Hickie and Rogers, 2011; Medvedev, 2017). Furthermore, its complex “biased signal transduction” property on the MT2/5-HT receptor heterodimer makes it difficult to isolate specific circadian regulatory effects from broader neurobiological impacts (Kamal et al., 2015). Agomelatine’s translational relevance to platelet function is particularly intriguing because serotonin is a potent platelet agonist (Kamal et al., 2015). Its dual modulation of the melatonergic and serotonergic systems offers a unique molecular avenue to counteract stress-induced platelet hyperactivity, making it a promising candidate for exploring the neuro-cardiovascular axis where mood disorders intersect with thrombotic risks (Shimohina et al., 2015) (**Table 1**).

### 5.4 SR9009

SR9009 is a synthetic REV-ERBα/β agonist designed to regulate the stability of the molecular clock by inhibiting the expression of core circadian genes such as *Bmal1* (Yang et al., 2025). Currently, the clinical status of SR9009 is strictly limited to the preclinical stage, with evidence derived solely from *in vivo* animal models and cellular assays that demonstrate its ability to alleviate continuous light-induced circadian disorders, improve metabolic dysfunction, and exert neuroprotective effects (Sulli et al., 2018; Yang et al., 2025). The absolute lack of human clinical trials means its safety profile and side effects in humans are entirely unknown. A major limitation hindering its translational progress is the documentation of off-target effects, as SR9009 has been shown to affect metabolism and cellular proliferation independently of the REV-ERB pathway, suggesting potential non-specific toxicities (Dierickx et al., 2019). Nevertheless, SR9009 holds the most direct translational relevance to platelet biology among current candidates. Since REV-ERBα is the principal driver of the morning surge in platelet activation and thrombosis, structurally refining SR9009 to eliminate off-target effects could yield a highly specific chrono-pharmacological agent capable of preventing morning-onset acute myocardial infarctions (Shi et al., 2022) (**Table 1**).

### 5.5 VTP-43742

VTP-43742 is an inverse agonist/antagonist targeting the nuclear receptor RORγ, which is involved in both inflammatory responses and the direct transcriptional regulation of circadian clock genes such as ARNTL/Bmal1, PER2, and CRY1 (Takeda et al., 2012). VTP-43742 and its derivatives remain in the experimental and preclinical stages, primarily investigated in the context of autoimmune diseases and tumor treatment (Kojetin and Burris, 2014). A significant limitation of this drug is its profound tissue- and genetic-context selectivity, meaning that modulators of RORγ can exhibit differentiated or even diametrically opposite effects depending on the cell type, which severely limits systemic administration safety. Translationally, by directly suppressing RORγ, VTP-43742 modulates the upstream core clock regulators (Kojetin and Burris, 2014). If tissue-specific delivery systems can be developed, manipulating the RORγ-circadian axis could indirectly regulate megakaryocyte thrombopoiesis and the baseline functional state of circulating platelets, offering a novel intervention for autoimmune-mediated thrombotic disorders (Shi et al., 2022) (**Table 1**).

### 5.6 PF-670462

PF-670462 is a highly selective inhibitor of Casein Kinase 1/(CK1δ/ϵ), a central kinase complex responsible for the phosphorylation and degradation of the core clock protein PER (Walton et al., 2009). Its application is currently restricted to preclinical research, with evidence from extensive animal models demonstrating that PF-670462 effectively prolongs the circadian rhythm cycle and restores rhythmic amplitude in chronodisrupted disease models (Meng et al., 2010). A critical limitation of PF-670462 is that its physiological and rhythmic phase-delay effects are heavily dose- and dosing-time-dependent, and are highly susceptible to environmental photoperiods, which can partially counteract the drug’s efficacy (Kim et al., 2013). Furthermore, its broad regulatory capability over both central and peripheral tissues complicates targeted therapy without causing systemic chronodisruption (Meng et al., 2010). Translationally, PF-670462’s ability to rapidly delay clock gene expression in peripheral tissues suggests it could be utilized to reset the independent clocks of circulating platelets or bone marrow megakaryocytes (Shi et al., 2022). Carefully timed administration could theoretically shift the peak of platelet aggregability away from the vulnerable early morning hours, providing a biochemical safeguard against ischemic cardiovascular events (Shi et al., 2022) (**Table 1**).

### 5.7 Critical comparison and translational relevance

While targeting the circadian rhythm presents a novel therapeutic frontier, there is a distinct dichotomy in the clinical status and translational readiness of the discussed pharmacological agents. Melatonin receptor agonists (tasimelteon, ramelteon, agomelatine) possess the highest level of clinical evidence, being FDA- and EMA-approved for sleep and mood disorders (Dhillon and Clarke, 2014). They exhibit favorable safety profiles, lacking the addiction or withdrawal risks associated with traditional sedatives, though they are limited by mild side effects (Pandi-Perumal et al., 2011) such as headaches or transient liver enzyme elevations (as seen with tasimelteon). Translationally, because melatonin directly modulates platelet activity via platelet-surface receptors, these approved agonists hold immediate repurposing potential for managing circadian-driven platelet hyperactivity in cardiovascular patients (Li et al., 1997). Conversely, core clock gene modulators (SR9009, VTP-43742, PF-670462) remain strictly in the preclinical and experimental stages (Solt et al., 2012). SR9009, despite its high relevance to platelet biology due to its direct action on REV-ERBα (the primary driver of the morning surge in platelet activation and thrombosis) (Muller et al., 1989), lacks human clinical trial data. Furthermore, its translational progress is severely limited by documented off-target effects and metabolic alterations that occur independently of REV-ERB pathways (Dierickx et al., 2019). Similarly, the CK1δ/ϵ inhibitor PF-670462 is restricted to animal models (Sprouse et al., 2009), and its rhythmic regulatory effects are highly susceptible to environmental photoperiod changes, complicating dosage and timing in human applications[ (Kim et al., 2013). Therefore, while melatonin agonists offer immediate, safe, but indirect regulation of platelet circadian rhythms, direct molecular clock targets (like REV-ERBα and RORγ) provide greater specificity for thrombosis prevention but require significant optimization to overcome preclinical toxicity and off-target limitations (Kojetin and Burris, 2014) (**Table 1**).

**Table 1:** Critical comparison of circadian rhythm-targeting drugs and their translational relevance.

Drug name	Molecular target	Clinical status/evidence level	Documented side effects	Key limitations in clinical translation	Translational relevance to platelets	Reference
Aspirin	COX-1/Gut Microbiota Modulato	Clinically Approved; High-level RCT evidence for bedtime dosing	Gastrointestinal bleeding, mucosal ulceration	nter-individual variation in overnight thrombopoiesis rates	Maximizes inhibition of overnight-shed platelets; blunts morning TXA2 surge	(Tofler et al., 1987; Von Kanel et al., 2004; Hermida et al., 2007)
Clopidogrel	P2Y12 Receptor	Clinically Approved; Moderate-level chronotherapeutic evidence	Hemorrhage, rash, neutropenia (rare)	Variable hepatic CYP metabolism; subject to CYP2C19 polymorphisms	Evening dosing optimizes active metabolite generation via clock-controlled CYP pathways	(Pagani et al., 2010; Vandenberghe et al., 2022)
Tasimelteon	Dual MT1/MT2 Agonist	FDA/EMA Approved (Non-24-hour sleep-wake disorder)	Headache, elevated alanine aminotransferase, nightmares	High cost; lack of large-scale head-to-head trials against melatonin	Resynchronizes central SCN pacemaker; indirectly dampens HPA-mediated morning platelet surge	(Dhillon and Clarke, 2014; Lavedan et al., 2015; Leger et al., 2015; Lockley et al., 2015; Nishimon et al., 2019)
Ramelteon	Selective MT1/MT2 Agonist	FDA Approved (Insomnia)	Somnolence, dizziness, fatigue, decreased testosterone	Short half-life (1–2 hours); low oral bioavailability due to first-pass metabolism	Directly attenuates surface-expressed melatonin receptor pathways on platelets to decrease aggregation	(Kato et al., 2005; Richardson et al., 2008; Miyamoto, 2009; Pandi-Perumal et al., 2011; Mishima, 2012; Saharov et al., 2025)
SR9009	Rev-Erbα/β Agonist	Preclinical Stage (*In vitro*/*In vivo* animal models)	Potential metabolic toxicity, cell viability issues in high doses	Exceptionally poor oral bioavailability; unknown human safety profile	Directly dampens the Nr1d1-mediated OPHN-1/RhoA/ERM pro-thrombotic cascade	(Sulli et al., 2018; Yang et al., 2025)

## 6 Critical evaluation, conflicting findings, and knowledge gaps

### 6.1 Conflicting evidence on circadian drivers

A major point of contention in the field is the relative contribution of the intrinsic platelet/megakaryocyte molecular clock versus systemic circadian signals (Scheer et al., 2011). While substantial evidence points to intrinsic regulators like Rev-erbα as the primary drivers of platelet morning hyperreactivity (Shi et al., 2022), conflicting studies argue that systemic neuroendocrine rhythms such as the morning surge in catecholamines, cortisol, and intrinsic vascular tone are the dominant forces dictating platelet aggregation (Tofler et al., 1987). For instance, some *in vivo* models suggest that genetic ablation of core clock genes (e.g., Bmal1) in megakaryocytes disrupts platelet rhythms entirely (Zhao et al., 2011), whereas other studies demonstrate that systemic stress hormones can override these local molecular clocks and induce platelet activation regardless of the intrinsic TTFL status (Morris et al., 2012). The exact hierarchical relationship between central SCN signals, rhythmic gut microbiome metabolites, and the intrinsic megakaryocyte clock remains a critical knowledge gap (Bautista et al., 2025b).

### 6.2 Limitations of current experimental models

A significant limitation in circadian platelet research is the profound reliance on murine models. Mice are strictly nocturnal animals, meaning their behavioral, metabolic, and cardiovascular circadian phases are completely inverted compared to diurnal humans (Feuring et al., 2009; Scheer et al., 2011; Esposito et al., 2020). Consequently, translating the temporal dynamics of platelet activation, thrombus formation, and chronotherapy efficacy from mice to humans requires cautious interpretation (D'souza et al., 2021; Ruan et al., 2021; Lecour et al., 2022). Furthermore, most *in vitro* studies evaluating platelet aggregability rely on isolated platelet-rich plasma or washed platelets, fundamentally stripping them of their natural, dynamic microenvironment. These static assays fail to account for rhythmic interactions with the endothelium, rhythmic blood flow shear stress, and diurnal fluctuations in circulating immune cells, which may artificially amplify or mask intrinsic platelet rhythms (Hartley et al., 2008; Somanath et al., 2011; Hearn et al., 2023).

### 6.3 Translational and clinical knowledge gaps

Clinically, there is a distinct gap between biochemical findings and clinical outcomes regarding chronotherapy. While laboratory evidence overwhelmingly supports that evening administration of antiplatelet agents (such as low-dose aspirin or clopidogrel) significantly blunts the morning surge in platelet reactivity (Buurma et al., 2019; Maki and Duell, 2024), large-scale randomized controlled trials have yielded conflicting results regarding whether this translates to a significant reduction in hard clinical endpoints, such as Major Adverse Cardiovascular Events (MACE) or overall mortality (Bowman et al., 2018).

Additionally, the current literature heavily focuses on healthy physiological states, leaving a massive void regarding how aging, sex differences, and chronic comorbidities influence platelet circadian rhythms. Aging itself is universally associated with a progressive decay in peripheral clock coordination and dampening of circadian amplitudes (Pagani et al., 2010; Vandenberghe et al., 2022). Similarly, distinct sexual dimorphisms exist in both baseline platelet reactivity and the expression kinetics of clock target genes, which remain poorly characterized in clinical cohorts (Thosar et al., 2018). Furthermore, metabolic diseases, such as diabetes and severe obesity, are known to “flatten” or disrupt the amplitude of circadian oscillations (Scheer et al., 2011; Schrader et al., 2024). Whether the circadian targets discussed (e.g., Rev-erbα, MT1, MT2 receptors) remain viable therapeutic options in patients whose central or peripheral clocks are already severely uncoupled remains largely unknown (Thaiss et al., 2014).

## 7 Conclusion

This review establishes the pivotal role of the platelet circadian rhythm in both physiology and pathology. Phenomenologically, platelet activation and aggregation peak in the morning, coinciding with the well-documented morning surge of major adverse cardiovascular events such as myocardial infarction and ischemic stroke. Mechanistically, the intrinsic molecular clock particularly Rev-erbα drives platelet hyper-reactivity through the OPHN-1/RhoA/ERM cascade. This core mechanism is further fine-tuned by neuroendocrine signals (catecholamines, cortisol), melatonin signaling, and gut microbial metabolites (e.g., short-chain fatty acids, 5-HT, S1P), which intersect with the canonical TTFL clock genes (*Clock, Bmal1, Per, Cry*). Disease relevance: In cardiovascular disease, Rev-erbα-mediated platelet activation promotes arterial thrombosis; in neurological diseases, platelets act as inflammatory mediators and peripheral biomarkers contributing to neurodegeneration; in metabolic diseases, circadian disruption aggravates insulin resistance, chronic low-grade inflammation, and mitochondrial dysfunction, thereby exacerbating metabolic syndrome. Therapeutic strategies: Melatonin receptor agonists (tasimelteon, ramelteon, agomelatine) are already approved for sleep–wake disorders, exhibit favorable safety profiles, and hold translational potential for modulating platelet activity. In contrast, direct core-clock modulators (e.g., SR9009 targeting Rev-erbα, PF-670462 targeting CK1δ/ϵ) have demonstrated anti-thrombotic effects in animal models but lack human data and show off-target effects. Future directions: Key challenges include reconciling phase differences between nocturnal rodent models and diurnal humans, elucidating the origin of clock proteins in anucleate platelets (megakaryocyte inheritance vs. non-transcriptional redox oscillations), and resolving inconsistent clinical endpoint evidence. Personalized chronotherapy based on individual circadian endophenotypes represents the next frontier for translating platelet circadian biology into clinical practice.

## 8 Discussion

This review highlights that platelets are not only the executors of hemostasis and thrombosis but also a vital bridge connecting circadian rhythms with systemic cardiovascular, neurological, and metabolic diseases. The circadian network coordinates platelet activation and immune function through core clock genes and peripheral tissue clocks, establishing a strong theoretical basis for chronotherapy. However, a critical evaluation of the current literature reveals significant gaps and limitations that must be addressed before these findings can be successfully translated into clinical guidelines.

A primary limitation is the heavy reliance on nocturnal murine models, which inherently complicates the translation of rhythmic phase findings to diurnal human. Moreover, there is conflicting evidence regarding the hierarchy of circadian control in platelets, while intrinsic molecular components like Rev-erbα strongly dictate platelet hyperreactivity, the extent to which these intrinsic clocks can be overridden by systemic neuroendocrine surges (e.g., cortisol, catecholamines) or rhythmic microbiome metabolites remains debated.

Crucially, from a molecular perspective, because circulating platelets are anucleate, they cannot undergo active transcription. This means their daily oscillations rely either on the translational inheritance of clock proteins and messenger RNAs from their nucleated precursor megakaryocytes during thrombopoiesis, or on non-transcriptional metabolic oscillators such as peroxiredoxin (Prx) redox cycles that persist autonomously in the cytoplasm. Delineating the exact half-life, stability, and degradation kinetics of clock proteins within the anucleate environment of circulating blood cells versus their genomic regulation in bone marrow megakaryocytes remains a foundational challenge for the field.

Clinically, while evening-dosed antiplatelet chronotherapy successfully dampens the morning biochemical surge in platelet aggregation, large-scale evidence remains inconsistent regarding its ability to decisively reduce hard cardiovascular endpoints compared to standard morning dosing. Future research directions must focus on overcoming these limitations. Future clinical trials must systematically investigate how demographic factors, aging, sex differences, and chronic metabolic comorbidities which inherently blunt circadian amplitudes alter the downstream efficacy of circadian-targeted therapies (Scheer et al., 2011). By transitioning from highly controlled *in vitro* assays to dynamic, *in vivo* humanized models, and leveraging artificial intelligence and machine learning pipelines for personalized chrono-pharmacology, we can optimize dosing schedules based on an individual’s unique circadian endophenotype. Ultimately, unlocking these mechanisms will allow clinicians to fully translate the circadian rhythm of platelets into precise, time-sensitive therapeutic strategies that maximize cardioprotection while minimizing bleeding risks.
